# Factors associated with energy intake at 28 days in preterm infants: a prospective cohort study

**DOI:** 10.1007/s00431-026-07529-7

**Published:** 2026-10-23

**Authors:** Jimmy Kok-Foo Lee, Shed-Peng Khor, Jeevitha Mariapun, Aliffah Zakaria, Huey-Ling Chow, Nisah Abdullah, Mohd Nizam Mat Bah, Jodi Woan-Fei Law, Learn-Han Lee, Amutha Ramadas, Vengadesh Letchumanan

**Affiliations:** 1 Clinical School Johor Bahru, Jeffrey Cheah School of Medicine and Health Sciences, Monash University Malaysia , No. 8, Masjid Sultan Abu Bakar, Johor Bahru, Johor 80100 Malaysia; 2 JCSMHS, Monash University Malaysia, Jeffrey Cheah School of Medicine and Health Sciences, Monash University Malaysia, Bandar Sunway, Selangor Malaysia; 3 Department of Paediatrics, Hospital Sultanah Aminah, Johor Bahru, Malaysia; 4 Microbiome Research Group, Research Centre for Life Science and Healthcare, Nottingham Ningbo China Beacons of Excellence Research and Innovation Institute, University of Nottingham Ningbo China, Ningbo, China; 5 Pathogen Resistome Virulome and Diagnostic Research Group, Jeffrey Cheah School of Medicine and Health Sciences, Monash University Malaysia, Bandar Sunway , Selangor Malaysia

**Keywords:** Preterm infants, Energy intake, Early postnatal growth faltering, Bronchopulmonary dysplasia, Necrotising enterocolitis

## Abstract

Early energy intake is critical for growth in preterm infants, but evidence on factors affecting energy delivery in middle-income settings is limited. This study identified factors associated with energy intake at 28 days in preterm infants. Infants with birthweight ≤ 1500 g and gestational age < 33 weeks surviving to 28 days were enrolled. Serial energy and protein intake, growth and morbidities were assessed. Multiple linear and mixed-effects regression evaluated energy intake at 28 days and longitudinally, respectively. Among 168 infants, mean birthweight was 1113.5 (SD 241.9) g and gestational age 29.3 (SD 2.1) weeks. Bronchopulmonary dysplasia (BPD) occurred in 31.9% and necrotising enterocolitis (NEC) in 4.8%. Full enteral feeding and energy intake of 100 kcal/kg/day were achieved at 16.8 (SD 9.3) and 17.2 (SD 7.4) days, respectively. Energy and protein intake from parenteral and enteral nutrition remained below recommended targets during the first 14 days, while the protein-energy ratio remained inadequate through day 28. Mean growth velocity to 28 days was 5.1 (SD 3.6) g/kg/day, with a median weight z-score change of − 1.48 (IQR − 1.78 to − 1.27). After adjustment, energy intake at 28 days was lower in infants with BPD (mean difference − 11.7 kcal/kg/day, 95% CI − 23.1 to − 0.4) and NEC (− 33.3 kcal/kg/day, 95% CI − 57.3 to − 9.3). *Conclusion*: Preterm infants experienced suboptimal early energy and protein intake and growth. BPD and NEC were independently associated with lower energy intake at 28 days, highlighting ongoing nutritional challenges, particularly in preterm infants with major morbidities.

**Table:** 

**What is Known:** *• Optimal early postnatal growth remains challenging to attain in sick preterm infants.* *• Recommended energy and protein targets may remain unmet despite structured nutrition protocols.*	
**What is New:** *• Lower energy intake at 28 days was independently associated with NEC and subsequent development of BPD.* *• Suboptimal parenteral and enteral nutrient delivery, particularly during the first 14 days, occurred alongside subsequent growth faltering.*

**Supplementary Information:**

The online version contains supplementary material available at https://doi.org/10.1007/s00431-026-07529-7.

## Introduction

Preterm birth interrupts the third trimester, the period of greatest foetal weight gain [1]. Loss of this critical phase of in utero growth, combined with early postnatal growth faltering, contributes to adverse long-term developmental outcomes [2]. Preterm infants are nutritionally vulnerable [3] and require simultaneous initiation of parenteral nutrition (PN) [4] and advancement of enteral nutrition (EN) soon after birth [5]. International guidelines recommend energy intakes of 90–120 kcal/kg/day via PN or 110–135 kcal/kg/day via EN [4] to support intrauterine-equivalent growth of approximately 15 g/kg/day [6], together with protein intake of 2.5–3.5 g/kg/day [7] and a protein–energy ratio of 3.2–4.1 g/100 kcal [8]. Achieving these targets in clinical practice remains challenging [3].

Energy and protein intake are influenced by fluid management strategies and neonatal morbidities, which vary across neonatal units. Fluid regimens typically increase from 60–100 mL/kg/day on day 1 to 100–180 mL/kg/day by days 4–5 [9, 10], with full enteral volumes ranging from 140 to 200 mL/kg/day [11]. Fluid restriction may constrain nutrient delivery but has been used to reduce the risks of patent ductus arteriosus (PDA) and necrotising enterocolitis (NEC) [12]. Excessive fluid administration predisposes unstable infants to haemodynamically significant patent ductus arteriosus (hsPDA) [13] and increases the risks of intraventricular haemorrhage (IVH), bronchopulmonary dysplasia (BPD), NEC, and mortality [14]. Nutritional intake is also influenced by local protocols [15], early respiratory status [16], and morbidities including hsPDA [17], BPD [18], and NEC.

Most evidence on early nutrition in very preterm (VP) infants comes from high-income countries [9, 19–24], with limited data from middle-income settings [25]. Few studies have examined factors influencing early energy intake, particularly the associations with major morbidities. This study evaluated energy and protein intake over the first 28 days of life and identified factors associated with energy intake at 28 days in a middle-income setting.

## Materials and methods

### Study design and setting

A prospective cohort study was conducted at a regional Level IIIB neonatal centre in Malaysia. Preterm infants were recruited over 14 months (2020–2021). The study was registered with the National Medical Research Registry (NMRR-19–3773-52331 IIR).

### Participants

Eligible infants had a birthweight ≤ 1500 g, gestational age < 33 weeks and survived to 28 days of life. Gestational age was determined using obstetric dating or the Ballard score (accuracy ± 2 weeks) [26]. Exclusion criteria were death before admission, trisomy syndromes, and major congenital anomalies.

### Sample size calculation

Using a pilot standard deviation of 25 kcal/kg/day, sample sizes were calculated to detect differences in mean energy intake according to BPD (30% incidence, Δ = 10 kcal/kg/day) and NEC (5% incidence, Δ = 30 kcal/kg/day) at *α* = 0.05 and 80% power. Estimated sample sizes were 234 for BPD and 115 for NEC analyses, and the larger sample size was the study target.

### Data collection and clinical variables

Demographic, perinatal, and clinical variables were prospectively recorded. Morbidities included NEC (Bell’s stage ≥ 2), early-onset sepsis (≤ 72 h), late-onset sepsis (> 72 h), BPD (oxygen requirement for at least 28 days and at 36 weeks’ postmenstrual age (PMA)), IVH (Papile classification), and hsPDA (ductal diameter > 1.5 mm with evidence of left-sided volume loading and a left atrium-to-aorta ratio > 1.5). Clinical Risk Index for Babies II (CRIB II) scores were recorded on admission [27]. Weight was measured twice weekly on a scale with 5-g resolution. Infants were categorised into ≤ 1000 g (G1) and 1005–1500 g (G2).

### Fluid and nutritional practices, and energy–protein calculations

Fluid management followed national guidelines with volumes of 60, 80, 100, 120, and 150 mL/kg/day on days 1, 2, 3, 4, and 7, respectively. Volumes were restricted to 120–135 mL/kg/day if echocardiography between days 5 and 7 confirmed hsPDA.

Starter PN contained 3.3 g amino acids per 100 mL, compared with 3.0 g in standard PN, while both formulations supplied 10 g glucose per 100 mL. Lipids (0.178 g/mL) were initiated by day 2 at 1 g/kg/day, with amino acids and lipids increased by 0.5 g/kg/day to targets of 3.5 g/kg/day and 3 g/kg/day, respectively. Expressed breast milk was used for trophic feeds and advanced by 10–20 mL/kg/day as tolerated. Human milk fortifier was added once EN exceeded 100 mL/kg/day. Full enteral feeding was defined according to local practice as enteral intake ≥ 100 mL/kg/day, after which PN was discontinued.

Energy and protein intake from PN and EN were calculated on days 1, 3, 5, 7, 10, 14, 21, and 28. PN provided 4 kcal/g for glucose, 4 kcal/g for amino acids, and 1.8 kcal/mL for lipids. Preterm formula, breast milk, and fortified breast milk provided 24, 20, and 24 kcal per 30 mL, with protein contents of 2.3, 1.5, and 2.35 g per 100 mL, respectively [24].

### Growth assessment

Weight z-scores were calculated using PediTools® [28]. Growth velocity was derived using exponential and two-point models [29], with birthweight as the baseline.

### Outcome measures

The primary outcome was total energy intake at 28 days of life. Secondary outcomes included the proportion of energy derived from EN and PN, the protein–energy ratio and time to reach 100 kcal/kg/day. Feeding progression measures included timing of first enteral feed and achievement of full enteral feeding. Growth outcomes included time to regain birthweight, growth velocity using exponential and two-point models and changes in weight z-scores from birth to 28 days and to 36 weeks’ PMA.

### Statistical analysis

Normality was assessed with the Kolmogorov–Smirnov test. Continuous variables were compared using the *t*-test or Mann–Whitney *U* test, and categorical variables using the chi-square or Fisher’s exact test. Variables with *p* < 0.25 in univariable analysis were included in multiple linear regression to identify factors associated with energy intake at 28 days. Serial energy intake was analysed with multilevel mixed-effects regression. Analyses were performed using available cases, with no imputation for missing data. Model assumptions were checked using residual diagnostics and variance inflation factors. Analyses were performed in Stata version 16.1 (StataCorp) with *p* < 0.05 deemed significant.

### Ethics approval and consent to participate

Ethical approval was obtained from the Medical Research Ethics Committee (MREC), Ministry of Health Malaysia, and the Monash University Human Research Ethics Committee (MUHREC Project 23588). Data were anonymised before analysis.

### Patient and public involvement

Parents and the public were not involved in the design, conduct or reporting of this study.

## Results

### Study population

**Fig. 1:**
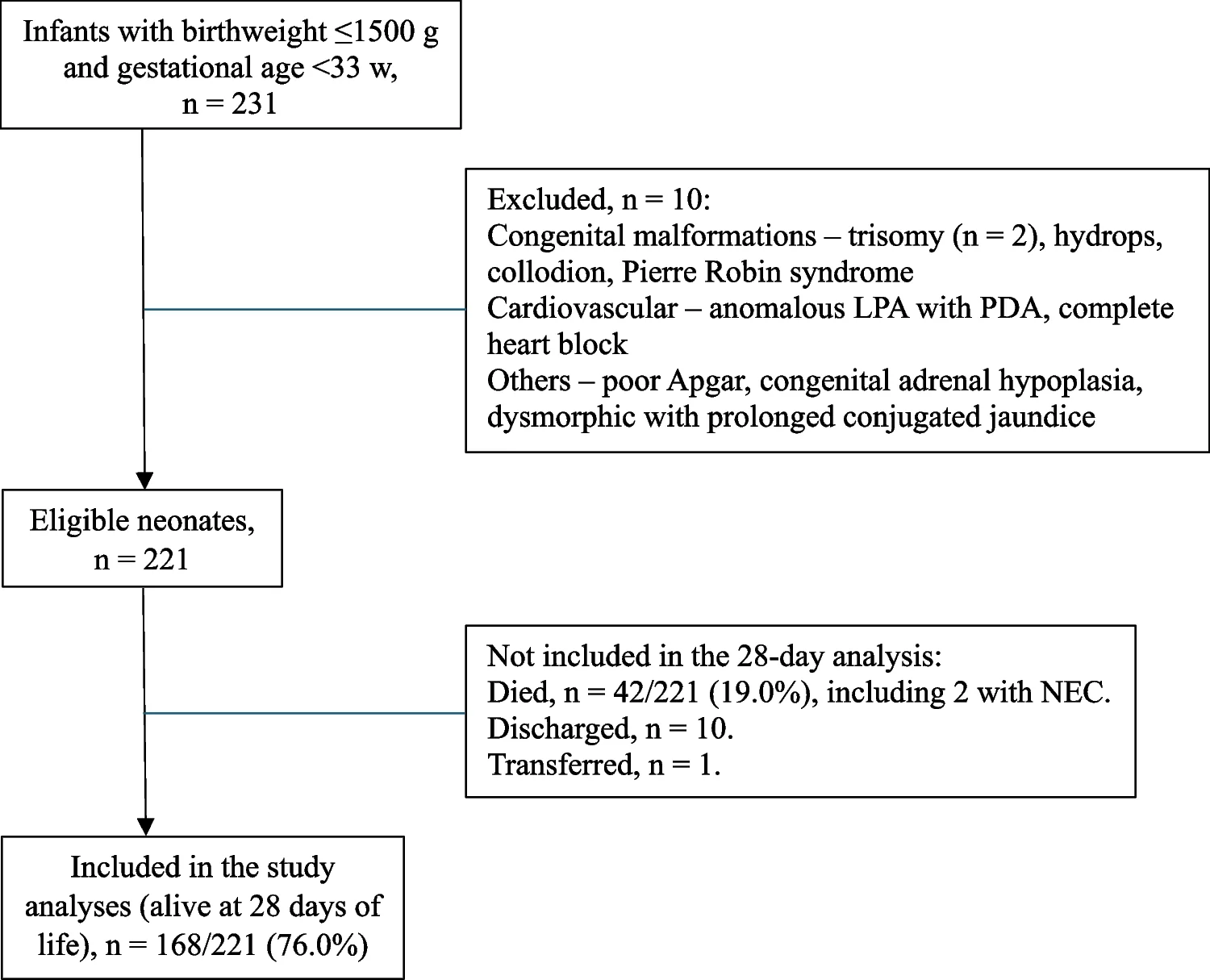
Flow diagram of participant recruitment and inclusion in the nutritional and growth analyses

Among 221 eligible infants, 168 (76.0%) survived to 28 days and were included in the analysis (Fig. 1). Infants ≤ 1000 g had lower admission temperatures, higher CRIB II scores, and longer durations of continuous positive airway pressure (CPAP) and mechanical ventilation than those 1005–1500 g (Table 1). The incidence of subsequent BPD was 31.9% and severe IVH 6.3%, both more frequent in the ≤ 1000 g group. Eight infants developed NEC (4.8%), three of whom died after 28 days of life.

**Table 1:** Demographic characteristics, neonatal events and morbidities by birthweight category (*n* = 168)

Parameter	Overall *n* = 168, unless specified	G1 (birthweight ≤ 1000 g) *n* = 56, unless specified	G2 (birthweight 1005–1500 g) *n* = 112, unless specified	*p*
Birthweight (g)				
Mean (SD) Median (IQR)	1113.5 (241.9) 1120 (950 to 1322.5)	835.5 (131.5) 875 (730 to 950)	1252.5 (144.8) 1250 (1120 to 1370)	
Gestational age (w),				
Mean (SD) Median (IQR)	29.3 (2.1) 29 (28 to 31)	27.4 (1.8) 28 (26 to 28)	30.2 (1.5) 30 (29 to 31.5)	< 0.001** < 0.001**
Sex (male)–*n* (%)	74 (44.0%)	19 (33.9%)	55 (49.1%)	0.062
Post-28-day mortality, * n* (%)	5 (3.0%)	4 (7.1%)	1 (0.9%)	0.025*
Admission temperature (℃), *n*	165	56	109	
Mean (SD)	34.76 (1.20)	34.47 (1.09)	34.94 (1.23)	0.026*
CRIB II scores, *n*	159	56	103	
Mean (SD)	7.50 (3.14)	10.57 (2.32)	5.83 (2.10)	< 0.001**
Duration of CPAP (days), mean (SD)	8.19 (11.69)	16.28 (14.32)	4.15 (7.37)	< 0.001**
Duration of ventilation (days)				
Overall, mean (SD)	7.21 (11.25)	13.29 (14.92)	4.17 (7.22)	< 0.001**
Survived till Discharge	*n* = 1636.34 (10.03)	*n* = 5211.42 (13.35)	*n* = 1113.96 (6.90)	< 0.001**
Died after 28 days	*n* = 535.54 (13.19)	*n* = 437.57 (14.30)	*n* = 127.42	0.800
Severe IVH (grade 3–4), *n*/*N* (%)	9/142 (6.3%)	7/55 (12.7%)	2/87 (2.3%)	0.013*
hsPDA, *n*/*N* (%)	39/157 (24.8%)	15/56 (26.8%)	24/101 (23.8%)	0.675
Pneumothorax, *n* (%)	3 (1.8%)	1 (1.8%)	2 (1.8%)	1.000
NEC, *n* (%)	8 (4.8%)	2 (3.6%)	6 (5.4%)	0.608
Died, *n*/*N* (%)	3/8 (37.5%)	2/2 (100%)	1/6 (16.7%)	
Sepsis episodes, *n* (%)	18 (10.7%)	6 (10.7%)	12 (10.7%)	1.000
Died, *n*/*N* (%)	1/18 (5.5%)	1/6 (16.7%)	0/12 (0%)	
EOS	3^ (none died)	0	3^	
LOS	16^ (1 died)	6 (1 died)	10^	
Subsequently developed BPD, *n*/*N* (%)	53/166 (31.9%)	34/54 (63.0%)	19/112 (17.0%)	< 0.001**

The 14 variables listed above were included in the univariable analysis of energy intake at 28 days of life. Four additional variables, namely ethnic group, parity, antenatal steroid exposure and place of birth, were also analysed but are not shown, as they did not differ significantly between birthweight groups

^*^Statistically significant (*p* < 0.05)

^**^ Statistically significant (*p* < 0.001)

^1 infant had both EOS and LOS

### Energy and protein intake

Total energy and protein intake increased progressively over 28 days (Table ST1). Both remained below recommended targets during the first 14 days. PN dominated energy and protein provision during the first week but declined as EN advanced (Figure SF1A–B). By around day 11, PN and EN contributed similar proportions of total energy and protein intake. Smaller infants had higher protein–energy ratios (PER) during the first week, with values remaining inadequate throughout the 28 days in both groups (Figure SF1C). PN-derived energy from amino acids and lipids remained below recommended targets during the first 14 days (Figure SF1D). Larger infants achieved significantly higher energy and protein intake than smaller infants at 28 days (Table ST1). Despite differences in illness severity, the mean age to reach 100 kcal/kg/day was 17.2 (SD 7.4) days with no between-group difference (Table 2). The mean age at initiation of enteral feeding (3.7 (SD 2.6) days) and the mean age at achievement of full enteral feeding (16.8 (SD 9.3) days) were also similar between the two groups.

**Table 2:** Age (days) at initiation of parenteral nutrition, first feed, attainment of full enteral feeding, achievement of 100 kcal/kg/day and birthweight regain in the energy and growth cohort (*n* = 168)

Variable	Overall *n* = 168, unless specified	G1 (birthweight ≤ 1000 g) *n* = 56, unless specified	G2 (birthweight 1005–1500 g) *n* = 112, unless specified	*p*
PN use, *n* (%)	141 (83.9%)	56 (100%)	85 (75.9%)	< 0.001**
Age at PN initiation (days),				
Mean (SD)	2.16 (3.27)	1.29 (1.43)	2.74 (3.95)	0.010*
Age at first feed (days),				
Mean (SD)	3.71 (2.55)	3.66 (1.40)	3.73 (2.97)	0.865
Age at full enteral feeding (days), *n*	167	55	112	
Mean (SD)	16.76 (9.32)	17.91 (10.73)	16.20 (8.54)	0.266
Age to 100 kcal/kg/day, *n*	167	55	112	
Mean (SD)	17.23 (7.39)	17.38 (8.12)	17.16 (7.05)	0.857
Age to regain birthweight (days),				
Mean (SD)	18.35 (7.24)	19.39 (8.15)	17.82 (6.85)	0.191

*Statistically significant (*p* < 0.05)

^**^Statistically significant (*p* < 0.001)

### Growth performance

The mean age to regain birthweight was 18.4 (SD 7.2) days, with no group difference (Table 2). Median weight z-score fell from –0.57 at birth to –1.99 at 28 days and –2.55 at 36 weeks’ PMA (Table 3). During the first 28 days, the median z-score change was –1.48 (IQR –1.78 to –1.27), with a greater decline in larger infants. At 36 weeks’ PMA, the median change in weight z-score from birth was –1.86 (IQR –2.31 to –1.43), with a greater decline among smaller infants who reached this stage later. Growth velocity across both intervals was markedly below the recommended 15 g/kg/day (Table 3). Larger infants achieved higher growth velocity to 28 days, while smaller infants showed greater gains by 36 weeks’ PMA.

**Table 3:** Growth status and growth velocity from birth to 28 days and from birth to 36 weeks’ postmenstrual age

Parameter	Overall *n* = 168, unless specified	G1 (birthweight ≤ 1000 g) *n* = 56, unless specified	G2 (birthweight 1005–1500 g) *n* = 112, unless specified	*p* value
**Growth status** (z-score and z-score difference)				
z-score				
Birth, median (IQR)	− 0.565 (− 1.19 to − 0.045)	− 0.545 (− 1.405 to − 0.045)	− 0.605 (− 1.15 to − 0.045)	0.708
28 days of life, median (IQR)	− 1.995 (− 2.63 to − 1.56)	− 1.86 (− 2.49 to − 1.54)	− 2.065 (− 2.655 to − 1.595)	0.252
36 weeks’ PMA, *n*	(*n* = 152)	(*n* = 54)	(*n* = 98)	
Median (IQR)	− 2.545 (− 2.93 to − 2.245)	− 2.795 (− 3.62 to − 2.39)	− 2.455 (− 2.77 to − 2.16)	< 0.003*
z-score difference				
Δz-score (birth to 28 days), *n*	(*n* = 168)	(*n* = 56)	(*n* = 112)	
Median (IQR)	− 1.48 (− 1.78 to − 1.27)	− 1.40 (− 1.61 to − 1.125)	− 1.54 (− 1.83 to − 1.33)	0.006*
Δz-score (birth to 36 weeks’ PMA), *n*	(*n* = 152)	(*n* = 54)	(*n* = 98)	
Median (IQR)	− 1.86 (− 2.39 to − 1.43)	− 2.37 (− 2.82 to − 1.86)	− 1.60 (− 2.04 to − 1.37)	< 0.001**
**Growth velocity** (g/kg/day) at selected time-points				
Birth to 28 days, *n*	(*n* = 168)	(*n* = 56)	(*n* = 112)	
Two-point, mean (SD)	5.09 (3.57)	4.08 (3.79)	5.60 (3.36)	0.009*
Exponential, mean (SD)	5.11 (3.60)	4.09 (3.81)	5.62 (3.39)	0.009*
Birth to 36 weeks’ PMA, *n*	(*n* = 151)	(*n* = 53)	(*n* = 98)	
Two-point, mean (SD)	8.60 (2.96)	9.76 (2.22)	7.97 (3.13)	< 0.001**
Exponential, mean (SD)	8.77 (3.10)	10.07 (2.40)	8.07 (3.22)	0.001*

^*^Statistically significant (*p* < 0.05)

^**^Statistically significant (*p* < 0.001)

### Factors associated with energy intake

Univariable analyses included six continuous variables (birthweight, gestational age, CPAP duration, duration of ventilation, admission temperature, and CRIB II score) and twelve categorical variables (ethnicity, parity, antenatal steroids, sex, place of birth, hsPDA, BPD, NEC, sepsis, IVH grade ≥ 3, mortality, and pneumothorax). Ten variables with *p* < 0.25 (birthweight, gestational age, CPAP duration, duration of ventilation, admission temperature, CRIB II score, BPD, NEC, sepsis, and IVH grade ≥ 3) were entered into the multivariable model (Table ST2). After adjustment, birthweight was not independently associated with total energy intake, while BPD and NEC were associated with adjusted mean differences of − 11.7 (95% CI − 23.1 to − 0.4) and − 33.3 (95% CI − 57.3 to − 9.3) kcal/kg/day at 28 days, respectively (Table 4). No significant interaction effects or multicollinearity were observed (Table ST3). Post hoc power was 52% for BPD and 77% for NEC. Multilevel mixed-effects regression analysed longitudinal energy intake by birthweight category and BPD/NEC status. NEC occurred in eight infants and BPD in 53, including one with both conditions, yielding 60 infants with BPD and/or NEC and 108 infants without BPD/NEC. Significant differences by birthweight were observed at week 4 (Figure SF2), whereas those by BPD/NEC status emerged at weeks 2, 3, and 4 (Figure SF3).

**Table 4:** Factors associated with total energy intake at 28 days of life

Variables	Simple linear regression		Multiple linear regression	
	B^a^ (95% CI)	*p*	B^b^ (95% CI)	*p*
Birthweight (g)^	0.019 (0.004 to 0.035)	0.017*	− 0.026 (− 0.056 to 0.004)	0.088
BPD (*n* = 53)	− 17.49 (− 25.28 to − 9.71)	< 0.001**	− 11.73 (− 23.09 to − 0.38)	0.043*
NEC stage ≥ 2 (*n* = 8)	− 18.08 (− 35.81 to − 0.35)	0.046*	− 33.26 (− 57.26 to − 9.30)	0.007*

*B*, unstandardised beta coefficient; ^a^Crude, ^b^Adjusted. The multiple linear regression included 137 complete cases. *Statistically significant (*p* < 0.05); **Statistically significant (*p* < 0.001)

^Comparison with categorical birthweight ≤ 1000 g:

Simple linear regression, coef − 13.068 (95% CI − 21.072 to − 5.065), *p* = 0.002;

Multiple linear regression, coef − 5.806 (95% CI − 18.592 to 6.980), *p* = 0.371

Model assumptions were met

There were no interactions among independent variables (Table ST3A), and no multicollinearity was detected (Table ST3B). The scatter plot of residuals versus predicted values showed no evidence of non-linearity or heteroscedasticity (violation of equal variance)

Adjusted *R*^2^ = 0.139

Final model equation (energy intake kcal/kg/day at 28 days of life):

= 169.0–33.3 NEC–11.7 BPD–15.5 IVH grade ≥ 3–5.9 sepsis–2.7 CRIB II + 1.7 admission temperature–2.0 gestational age–0.03 birthweight–0.3 duration of CPAP + 0.03 duration of ventilation

## Discussion

This study shows that early energy deficits were common within the fluid management strategy used in this cohort, with BPD and NEC independently associated with lower energy intake at 28 days. Most infants required over 2 weeks to reach 100 kcal/kg/day and early growth remained suboptimal, highlighting persistent challenges in achieving recommended energy–protein targets in similar neonatal settings.

The Malaysian National Neonatal Registry (MNNR) [30] reported 80.4% survival among infants < 32 weeks (500–1500 g), with BPD and NEC rates of 19.4% and 5.6%, respectively. In the parent study cohort (*n* = 221), BPD occurred in 29.4% and NEC in 4.5%. International NEC rates average 7% [31], and BPD has been reported in 23.5% of very low birthweight (VLBW) and 45.2% of extremely low birthweight (ELBW) infants [32]. PDA is reported in 57% of extremely preterm infants by day 7 [33]. In the present cohort, hsPDA was present in 24.8% of infants with available data during the first 28 days of life.

### Fluid policies and nutritional intake

The fluid management strategy used in this cohort provided the clinical context for early energy–protein delivery. The unit followed national fluid guidelines [34], comparable to protocols reported in developed Asian neonatal centres with favourable survival outcomes [10]. Higher volumes of 160–180 mL/kg/day by days 3–5, reported in several centres [9, 13, 35], were not used because of concerns regarding PDA, IVH, BPD, and NEC [36]. Infants with hsPDA received 120–135 mL/kg/day [17], with gradual escalation to 180 mL/kg/day only after PDA closure. Energy and protein intake remained below recommended targets during the first 14 days; however, the observational design does not allow these deficits to be attributed directly to the fluid strategy. Earlier advancement of enteral feeding may improve nutrient delivery, although the rate of advancement in clinical practice is influenced by clinical stability, concurrent morbidities, and feeding tolerance.

### Optimisation of parenteral and enteral nutrition

Energy and protein delivery in this cohort fell short of European Society for Paediatric Gastroenterology, Hepatology and Nutrition (ESPGHAN) recommendations [4, 7, 37]. Target intakes of 90–120 kcal/kg/day and 2.5–3.5 g protein/kg/day were not achieved during the first 14 days, and the protein–energy ratio remained below the optimal 3.2–4.1 g/100 kcal throughout the 28 days. PN is the predominant nutritional source during the first week for smaller infants [4]. Protein intakes of 2.0–3.6 g/kg/day are theoretically achievable with local PN formulations at fluid volumes of 60–120 mL/kg/day during the first four days of life. However, the glucose–amino acid mixture provides only 32–62 kcal/kg/day, making additional lipid- and milk-derived energy essential. Optimising early PN while advancing enteral milk feeding as tolerated may therefore improve nutrient delivery without unnecessarily prolonging PN. In this cohort, infants ≤ 1500 g and ≤ 1000 g reached 100 kcal/kg/day at 17.2 (SD 7.4) days and 17.4 (SD 8.1) days, respectively, later than the 11–14 days reported in VLBW cohorts [22, 38, 39] but similar to the 19–26 days reported in ELBW infants [24, 40].

### Nutritional challenges in BPD and NEC

Lower energy intake at 28 days was observed in infants who subsequently developed BPD and in those with NEC. Among eight infants with NEC, the median age at onset was 12.8 days (IQR 8.5–23.2; range 7–38); six developed NEC before day 28 and two thereafter. Earlier NEC necessitated interruption of enteral feeding [31, 41], reducing subsequent nutrient delivery, whereas later NEC illustrates the time-varying relationship between nutritional intake and morbidity. As BPD was diagnosed after day 28, its association with lower energy intake may reflect preceding respiratory illness, ventilatory requirements, hsPDA, and feeding difficulties rather than BPD itself. A secondary analysis of a randomised trial in extremely preterm infants reported differences in BPD severity between infants assigned to human milk fortification from day 3 versus day 14, suggesting a potential relationship between earlier enteral nutrient fortification and respiratory outcomes [42]. Malikiwi et al. [18] reported a growth velocity of 9.63 (SD 3.85) g/kg/day with a z-score change of –0.90. In contrast, this cohort had much lower growth velocity (3.70 (SD 3.78) g/kg/day) and a larger fall in weight z-score (–1.5, IQR –1.76 to –1.04), in the context of fluid restriction and morbidity.

### Comparison with studies using restrictive fluid regimens

Two Asian centres with similar fluid policies provide useful comparisons with the present cohort. Raturi et al. [24] reported a median growth velocity from birth to day 28 of 9.8 g/kg/day (IQR 1.70 to 28.12) in 144 ELBW infants. In a cohort of 236 VLBW infants, Lee et al. [25] found a mean growth velocity of –0.5 g/kg/day (95% CI − 1.3 to 0.3) in the first 14 days and 13.8 g/kg/day (95% CI 12.9 to 14.8) from days 14–28. The mean z-score decline was − 1.8 SD (95% CI − 2.0 to − 1.7) by 36 weeks’ PMA, comparable to the median decline of − 1.86 SD (IQR − 2.39 to − 1.43) observed in this cohort.

### Comparison with studies achieving nutritional targets

Several studies, including those involving ELBW cohorts, have demonstrated improved nutritional outcomes [20, 43]. Rossholt et al. [19] achieved full feeds by a median of 10 days despite high rates of BPD (48%) and PDA (45%), reporting a growth velocity of 15.8 g/kg/day and a z-score decline of only − 1.0. Two studies [20, 43] reported growth velocities > 15 g/kg/day from the second week onward, although these were calculated using the two-point method. Dejhalla et al. [20] reported that birthweight was regained by day 10, while Martin et al. [43] found that 75% remained small-for-gestational-age at 28 days. Larger VP/VLBW infants typically reached > 100 kcal/kg/day in 6–14 days [22, 44–46] and full feeds in 6–13 days [23, 44, 47–49].

Studies using updated ESPGHAN-aligned nutritional protocols have reported smaller declines in weight z-scores. Andrews et al. [9] reported a z-score change of − 0.27 SD (95% CI − 0.39 to − 0.15) at 36 weeks’ PMA among 396 infants who reached 180 mL/kg/day by day 7. Other studies have shown declines of − 0.4 to − 0.8 SD at 28 days [22, 49] and − 0.11 to − 0.66 SD at 36 weeks’ PMA [9, 47, 49]. Wittwer and Hascoet [21] reported improved outcomes after implementing revised ESPGHAN protocols. Under the older regimen, z-scores declined from − 1.6 at 28 days to − 1.8 at 56 days, whereas under the updated regimen, they remained stable at − 1.4. Although negative z-score changes persisted across studies, their magnitude was substantially smaller than that observed in the present cohort.

### Growth velocity and interpretation of z-scores

Growth velocity calculations vary across studies. The exponential method is preferred, but starting points differ, including birth to 28 days [49], birthweight regain to 36 weeks’ PMA [23] and weight nadir to 28 days [22]. Given differing gestational ages at birth, reporting growth velocity and z-score changes to 28 days and 36 weeks’ PMA provides more standardised comparisons.

### Clinical and policy implications

This study shows that preterm infants in a middle-income setting experienced suboptimal early energy and protein intake alongside marked early postnatal growth faltering. Improving outcomes may require optimising nutrient delivery within appropriate fluid volumes, advancing enteral milk feeding as tolerated, and sustaining efforts to reduce major morbidities. Prioritising early attainment of recommended nutritional targets may benefit similar settings, while recognising that negative z-score differences at 28 days and 36 weeks’ PMA persist across neonatal settings. Future prospective studies should evaluate nutritional strategies that optimise early nutrient delivery while accounting for time-varying relationships between nutritional intake, clinical instability, and major morbidities.

### Strengths and limitations

This study provides detailed local data on nutritional intake, morbidity, and early growth. Although limited by its single-centre and observational design, it highlights a persistent and clinically important gap relevant to NICUs with similar constraints. The analysis was limited to infants surviving to 28 days and may therefore underestimate the nutritional challenges among the sickest preterm infants who died earlier. Residual confounding by factors such as prematurity and illness severity cannot be excluded. The temporal relationships between BPD, NEC and nutritional intake warrant caution in interpreting these associations, while the small number of NEC cases further limits the certainty of the NEC findings.

## Conclusion

These findings highlight ongoing challenges in achieving adequate early energy and protein intake and growth in preterm infants in middle-income settings. Lower energy intake at 28 days was associated with NEC and subsequent development of BPD. Optimising early nutrient delivery within appropriate fluid volumes, advancing enteral milk feeding as tolerated, and sustaining improvements in clinical care may help address these nutritional challenges and reduce major morbidities.

## Supplementary Information

Below is the link to the electronic supplementary material.

ESM 1 (DOCX 239 KB)

## Data Availability

Data relevant to the study are included in the article and online supplementary material. Additional deidentified data maybe available from the corresponding author upon reasonable request, subject to applicable ethical and institutional requirements.

## Abbreviations

BPD: Bronchopulmonary dysplasia
CPAP: Continuous positive airway pressure
CRIB II: Clinical Risk Index for Babies II
ELBW: Extremely low birthweight
EN: Enteral nutrition
ESPGHAN: European Society for Paediatric Gastroenterology, Hepatology and Nutrition
hsPDA: Haemodynamically significant patent ductus arteriosus
IVH: Intraventricular haemorrhage
MNNR: Malaysian National Neonatal Registry
NEC: Necrotising enterocolitis
PDA: Patent ductus arteriosus
PER: Protein–energy ratio
PMA: Postmenstrual age
PN: Parenteral nutrition
VLBW: Very low birthweight
VP: Very preterm

## References

[1] Villar J, Cheikh Ismail L, Victora CG, Ohuma EO, Bertino E, Altman DG et al (2014) International standards for newborn weight, length, and head circumference by gestational age and sex: the Newborn Cross-Sectional Study of the INTERGROWTH-21st Project. Lancet 384(9946):857–868. 10.1016/S0140-6736(14)60932-6 25209487

[2] Gounaris AK, Sokou R, Gounari EA, Panagiotounakou P, Grivea IN (2023) Extrauterine growth restriction and optimal growth of very preterm neonates: state of the art. Nutrients. 10.3390/nu15143231 PMC10384682 37513649

[3] Embleton NE, Pang N, Cooke RJ (2001) Postnatal malnutrition and growth retardation: an inevitable consequence of current recommendations in preterm infants? Pediatrics 107(2):270–273. 10.1542/peds.107.2.270 11158457

[4] Joosten K, Embleton N, Yan W, Senterre T (2018) ESPGHAN/ESPEN/ESPR/CSPEN guidelines on pediatric parenteral nutrition: energy. Clin Nutr 37(6 Pt B):2309–14. 10.1016/j.clnu.2018.06.944 30078715

[5] Corpeleijn WE, Vermeulen MJ, van den Akker CH, van Goudoever JB (2011) Feeding very-low-birth-weight infants: our aspirations versus the reality in practice. Ann Nutr Metab 58(Suppl 1):20–9. 10.1159/000323384 21701164

[6] Aap C (1985) American Academy of Pediatrics Committee on Nutrition: nutritional needs of low-birth-weight infants. Pediatrics (Evanston) 75(5):976–986 3921937

[7] van Goudoever JB, Carnielli V, Darmaun D, Sainz de Pipaon M, nutrition EEECwgopp (2018) ESPGHAN/ESPEN/ESPR/CSPEN guidelines on pediatric parenteral nutrition: amino acids. Clin Nutr 37(6 Pt B):2315–23. 10.1016/j.clnu.2018.06.945 30100107

[8] Agostoni C, Buonocore G, Carnielli VP, De Curtis M, Darmaun D, Decsi T et al (2010) Enteral nutrient supply for preterm infants: commentary from the European Society of Paediatric Gastroenterology, Hepatology and Nutrition Committee on Nutrition. J Pediatr Gastroenterol Nutr 50(1):85–91. 10.1097/MPG.0b013e3181adaee0 19881390

[9] Andrews ET, Ashton JJ, Pearson F, Beattie RM, Johnson MJ (2019) Early postnatal growth failure in preterm infants is not inevitable. Arch Dis Child Fetal Neonatal Ed 104(3):F235–F241. 10.1136/archdischild-2018-315082 30135111

[10] Kusuda S (2019) Neonatal Intensive Care Manual for the infants born less than 28 weeks of gestation (ver 1.1), National Research Network of Japan

[11] Klingenberg C, Embleton ND, Jacobs SE, O’Connell LA, Kuschel CA (2012) Enteral feeding practices in very preterm infants: an international survey. Arch Dis Child Fetal Neonatal Ed 97(1):F56-61. 10.1136/adc.2010.204123 21856644

[12] Bell EF, Acarregui MJ (2014) Restricted versus liberal water intake for preventing morbidity and mortality in preterm infants. Cochrane Database Syst Rev 2014(12):CD000503. 10.1002/14651858.CD000503.pub3 PMC7038715 25473815

[13] Stephens BE, Gargus RA, Walden RV, Mance M, Nye J, McKinley L et al (2008) Fluid regimens in the first week of life may increase risk of patent ductus arteriosus in extremely low birth weight infants. J Perinatol 28(2):123–128. 10.1038/sj.jp.7211895 18046337

[14] Sellmer A, Bjerre JV, Schmidt MR, McNamara PJ, Hjortdal VE, Host B et al (2013) Morbidity and mortality in preterm neonates with patent ductus arteriosus on day 3. Arch Dis Child Fetal Neonatal Ed 98(6):F505–F510. 10.1136/archdischild-2013-303816 23893268

[15] Olsen IE, Richardson DK, Schmid CH, Ausman LM, Dwyer JT (2002) Intersite differences in weight growth velocity of extremely premature infants. Pediatrics 110(6):1125–1132. 10.1542/peds.110.6.1125 12456909

[16] Lee JK, Yu VY (1996) Calorie intake in sick versus respiratory stable very low birthweight babies. Acta Paediatr Jpn 38(5):449–454. 10.1111/j.1442-200x.1996.tb03525.x 8942002

[17] Hansson L, Lind T, Wiklund U, Ohlund I, Rydberg A (2019) Fluid restriction negatively affects energy intake and growth in very low birthweight infants with haemodynamically significant patent ductus arteriosus. Acta Paediatr 108(11):1985–1992. 10.1111/apa.14815 30980416

[18] Malikiwi AI, Lee YM, Davies-Tuck M, Wong FY (2019) Postnatal nutritional deficit is an independent predictor of bronchopulmonary dysplasia among extremely premature infants born at or less than 28 weeks gestation. Early Hum Dev 131:29–35. 10.1016/j.earlhumdev.2019.02.005 30825742

[19] Rossholt ME, Bratlie M, Wendel K, Aas MF, Gunnarsdottir G, Fugelseth D et al (2023) A standardized feeding protocol ensured recommended nutrient intakes and prevented growth faltering in preterm infants < 29 weeks gestation. Clin Nutr ESPEN 53:251–259. 10.1016/j.clnesp.2022.12.024 36657920

[20] Dejhalla M, Lahage N, Parvez B, Brumberg HL, La Gamma EF (2015) Early postnatal growth in a subset of convalescing extremely-low-birth-weight neonates: approximating the “index fetus” ex utero. J Pediatr Gastroenterol Nutr 61(3):361–366. 10.1097/MPG.0000000000000750 25651485

[21] Wittwer A, Hascoet JM (2020) Impact of introducing a standardized nutrition protocol on very premature infants’ growth and morbidity. PLoS One 15(5):e0232659. 10.1371/journal.pone.0232659 PMC7241817 32437423

[22] Roelants JA, Joosten KFM, van der Geest BMA, Hulst JM, Reiss IKM, Vermeulen MJ (2018) First week weight dip and reaching growth targets in early life in preterm infants. Clin Nutr 37(5):1526–33. 10.1016/j.clnu.2017.08.023 28912010

[23] Rochow N, Fusch G, Muhlinghaus A, Niesytto C, Straube S, Utzig N et al (2012) A nutritional program to improve outcome of very low birth weight infants. Clin Nutr 31(1):124–31. 10.1016/j.clnu.2011.07.004 21890250

[24] Raturi S, Zheng Q, Daniel LM, Shi L, Rajadurai VS, Agarwal PK (2017) Nutritional intake and growth velocity in preterm extremely low-birthweight infants in Asia: are we doing enough? J Paediatr Child Health 53(12):1199–1207. 10.1111/jpc.13630 28833725

[25] Lee LY, Lee J, Niduvaje K, Seah SS, Atmawidjaja RW, Cheah FC (2020) Nutritional therapies in the neonatal intensive care unit and post-natal growth outcomes of preterm very low birthweight Asian infants. J Paediatr Child Health 56(3):400–407. 10.1111/jpc.14634 31618507

[26] Lee AC, Panchal P, Folger L, Whelan H, Whelan R, Rosner B et al (2017) Diagnostic accuracy of neonatal assessment for gestational age determination: a systematic review. Pediatrics 140(6):e20171423. 10.1542/peds.2017-1423 29150458

[27] Parry G, Tucker J, Tarnow-Mordi W, Group UKNSSC (2003) CRIB II: an update of the clinical risk index for babies score. Lancet 361(9371):1789–91. 10.1016/S0140-6736(03)13397-1 12781540

[28] Chou JH, Roumiantsev S, Singh R (2020) PediTools electronic growth chart calculators: applications in clinical care, research, and quality improvement. J Med Internet Res 22(1):e16204. 10.2196/16204 PMC7058170 32012066

[29] Patel AL, Engstrom JL, Meier PP, Kimura RE (2005) Accuracy of methods for calculating postnatal growth velocity for extremely low birth weight infants. Pediatrics 116(6):1466–1473. 10.1542/peds.2004-1699 16322172

[30] Neoh SH and the MNNR Steering Committee (2020) Report of the Malaysian National Neonatal Registry 2020

[31] Neu J, Walker WA (2011) Necrotizing enterocolitis. N Engl J Med 364(3):255–264. 10.1056/NEJMra1005408 PMC3628622 21247316

[32] Lee SM, Sie L, Liu J, Profit J, Lee HC (2022) Evaluation of trends in bronchopulmonary dysplasia and respiratory support practice for very low birth weight infants: a population-based cohort study. J Pediatr 243:47-52 e2. 10.1016/j.jpeds.2021.11.049 PMC8960334 34838581

[33] Sung SI, Chang YS, Kim J, Choi JH, Ahn SY, Park WS (2019) Natural evolution of ductus arteriosus with noninterventional conservative management in extremely preterm infants born at 23-28 weeks of gestation. PLoS ONE 14(2):e0212256. 10.1371/journal.pone.0212256 PMC6374019 30759169

[34] Hussain NHP, Terence Thomas, Hishamshah (2019) Paediatric protocols for Malaysian hospitals, 4th edition

[35] Leaf A (2011) Guidelines for the Nutritional Care of Infants in the Neonatal Unit, University Hospital Southampton, updated 2014, accessed on 22 Oct 2022. University Hospital Southampton, Clinical Guidance

[36] Hamrick SEG, Sallmon H, Rose AT, Porras D, Shelton EL, Reese J et al (2020) Patent ductus arteriosus of the preterm infant. Pediatrics. 10.1542/peds.2020-1209 PMC7605084 33093140

[37] Moltu SJ, Bronsky J, Embleton N, Gerasimidis K, Indrio F, Koglmeier J et al (2021) Nutritional management of the critically Ill neonate: a position paper of the ESPGHAN committee on nutrition. J Pediatr Gastroenterol Nutr 73(2):274–289. 10.1097/MPG.0000000000003076 33605663

[38] Falciglia GH, Murthy K, Holl JL, Palac HL, Oumarbaeva Y, Woods DM et al (2018) Energy and protein intake during the transition from parenteral to enteral nutrition in infants of very low birth weight. J Pediatr 202:38-43.e1. 10.1016/j.jpeds.2018.07.010 30195557

[39] Han J, Zhang L, Li S, Zhang Y, Jiang Y, Chen X et al (2022) Associations of early nutrition with growth and body composition in very preterm infants: a prospective cohort study. Eur J Clin Nutr 76(1):103–110. 10.1038/s41430-021-00901-w 33790398

[40] Radmacher PG, Looney SW, Rafail ST, Adamkin DH (2003) Prediction of extrauterine growth retardation (EUGR) in VVLBW infants. J Perinatol 23(5):392–395. 10.1038/sj.jp.7210947 12847535

[41] Ou J, Courtney CM, Steinberger AE, Tecos ME, Warner BW (2020) Nutrition in necrotizing enterocolitis and following intestinal resection. Nutrients. 10.3390/nu12020520 PMC7071274 32085587

[42] Tucker MJ, Jeffcoat SH, Argent T, Travers CP, Salas AA (2025) Severity of bronchopulmonary dysplasia in infants born extremely preterm and randomized to early human milk fortification with a donor milk-derived fortifier for 2 weeks. J Pediatr 287:114750. 10.1016/j.jpeds.2025.114750 PMC12382184 40714049

[43] Martin CR, Brown YF, Ehrenkranz RA, O’Shea TM, Allred EN, Belfort MB et al (2009) Nutritional practices and growth velocity in the first month of life in extremely premature infants. Pediatrics 124(2):649–657. 10.1542/peds.2008-3258 PMC2859427 19651583

[44] Christmann V, Visser R, Engelkes M, de Grauw AM, van Goudoever JB, van Heijst AF (2013) The enigma to achieve normal postnatal growth in preterm infants--using parenteral or enteral nutrition? Acta Paediatr 102(5):471–479. 10.1111/apa.12188 23398476

[45] Dinerstein A, Nieto RM, Solana CL, Perez GP, Otheguy LE, Larguia AM (2006) Early and aggressive nutritional strategy (parenteral and enteral) decreases postnatal growth failure in very low birth weight infants. J Perinatol 26(7):436–42. 10.1038/sj.jp.7211539 16801958

[46] Izquierdo M, Martinez-Monseny AF, Pociello N, Gonzalez P, Del Rio R, Iriondo M et al (2016) Changes in parenteral nutrition during the first week of life influence early but not late postnatal growth in very low-birth-weight infants. Nutr Clin Pract 31(5):666–672. 10.1177/0884533616649582 27207940

[47] Behnke J, Estreich V, Oehmke F, Zimmer KP, Windhorst A, Ehrhardt H (2022) Compatibility of rapid enteral feeding advances and noninvasive ventilation in preterm infants-an observational study. Pediatr Pulmonol 57(5):1117–26. 10.1002/ppul.25868 35191216

[48] Maas C, Mitt S, Full A, Arand J, Bernhard W, Poets CF et al (2013) A historic cohort study on accelerated advancement of enteral feeding volumes in very premature infants. Neonatology 103(1):67–73. 10.1159/000342223 23095283

[49] Moltu SJ, Blakstad EW, Strommen K, Almaas AN, Nakstad B, Ronnestad A et al (2014) Enhanced feeding and diminished postnatal growth failure in very-low-birth-weight infants. J Pediatr Gastroenterol Nutr 58(3):344–351. 10.1097/MPG.0000000000000220 PMC3940525 24556755

