# Supplementary material for: Factors associated with energy intake at 28 days in preterm infants: a prospective cohort study
Source: Eur J Pediatr. 2026 Oct 23;185(11):881. doi: 10.1007/s00431-026-07529-7 (PMC13694520; doi:10.1007/s00431-026-07529-7)
Supplement: Supplementary file 1 [file 431_2026_7529_MOESM1_ESM.docx]

**Online Supplementary Material**

**Factors associated with energy intake at 28 days in preterm infants: a prospective cohort study**

Jimmy Lee et al.

**Contents**

Supplementary Figures (SF)

- Fig. SF1.

A. Mean energy intake from PN, EN and PN&EN (kcal/kg/day) at 1, 3, 5, 7, 10, 14, 21 and 28 days of life.
B. Mean protein intake from PN, EN and PN&EN (g protein/kg/day) at 1, 3, 5, 7, 10, 14, 21 and 28 days of life.
C. Protein-energy ratio (g protein/100 kcal) at 1, 3, 5, 7, 10, 14, 21 and 28 days of life between the two birthweight categories of infants.
D. Percentage contribution of glucose, amino acids and lipids to PN energy intake (kcal/kg/day) over 14 days of life.

- Fig. SF2. Mean energy intake (kcal/kg/day) at 7, 14, 21 and 28 days between G1 and G2 infants (error bars represent 95% confidence intervals).
- Fig. SF3. Mean energy intake (kcal/kg/day) at 7, 14, 21 and 28 days in infants with and without BPD/NEC (error bars represent 95% confidence intervals).

Supplementary Tables (ST)

- Table ST1. Total daily energy intake, total daily protein intake and protein–energy ratio (PER) at selected postnatal days (1–28) in the study cohort (n = 168).
- Table ST2. Multiple linear regression of the ten independent variables included in the analysis of energy intake at 28 days of life.
- Table ST3. Assessment of interaction terms and multicollinearity among factors associated with total energy intake at 28 days of life.

Figure SF1A-D. Longitudinal energy and protein intake patterns during the first 28 days of life in preterm infants.

**A** Mean energy intake from PN, EN and PN&EN (kcal/kg/day) at 1, 3, 5, 7, 10, 14, 21 and 28 days of life.

**B** Mean protein intake from PN, EN and PN&EN (g protein/kg/day) at 1, 3, 5, 7, 10, 14, 21 and 28 days of life.

**C** Protein-energy ratio (g protein/100 kcal) at 1, 3, 5, 7, 10, 14, 21 and 28 days of life between the two birthweight categories of infants.

**D** Percentage contribution of glucose, amino acids and lipids to PN energy intake (kcal/kg/day) over the first 14 days of life.

Figure SF2. Mean energy intake (kcal/kg/day) at 7, 14, 21 and 28 days between G1 and G2 infants (error bars represent 95% confidence intervals).

Figure SF3. Mean energy intake (kcal/kg/day) at 7, 14, 21 and 28 days in infants with and without BPD/NEC (error bars represent 95% confidence intervals).

Table ST1. Total daily energy intake, total daily protein intake and protein–energy ratio (PER) at selected postnatal days (1–28) in the study cohort (n = 168).

| **Variable at day of life** | **Overall**  **(n = 168)** | **G1 (Birthweight**  **≤1000 g)**  **(n = 56)** | **G2 (Birthweight**  **>1000–1500 g)**  **(n = 112)** | ***p*** |
| --- | --- | --- | --- | --- |
| **Total energy intake**  (kcal/kg/day), mean (SD)  1  3  5  7  7, median (IQR)*  10  14  14, median (IQR)*  21  21, median (IQR)*  28  28, median (IQR)* | 24.28 (4.64)  45.37 (9.77)  65.48 (12.56)  78.31 (14.84)  81.2 (69.2 to 88.6)  81.52 (16.86)  87.71 (18.73)  87.5 (75.0 to 100.2)  105.20 (22.22)  109.5 (89.1 to 120.1)  113.68 (25.01)  119.6 (97.0 to 131.6) | 22.33 (4.08)  44.81 (10.73)  64.93 (12.12)  79.47 (14.86)  80.3 (71.0 to 89.5)  81.01 (16.27)  85.07 (19.30)  86.8 (71.0 to 99.7)  101.04 (22.61)  99.3 (86.6 to 116.5)  104.80 (24.30)  105.8 (84.5 to 124.3) | 25.25 (4.62)  45.64 (9.30)  65.76 (12.82)  77.73 (14.86)  81.2 (66.7 to 87.9)  81.78 (17.21)  89.03 (18.39)  87.9 (76.6 to 101.1)  107.28 (21.83)  112.1 (91.2-121.1)  118.11 (24.26)  124.8 (103.2 to 134.2) | **<0.001**  0.605  0.689  0.475  0.475  0.782  0.197  0.333  0.086  **0.048**  **0.001**  **0.001** |
| **Total daily protein intake**  (g/kg/day), mean (SD)  1  3  5  7  10  14  21  28 | 0.28 (0.42)  1.20 (0.98)  1.78 (1.15)  2.13 (1.11)  2.00 (1.06)  2.14 (1.01)  2.71 (0.98)  3.00 (1.07) | 0.40 (0.43)  1.41 (0.96)  2.03 (1.05)  2.30 (0.94)  1.99 (1.01)  2.09 (0.99)  2.61 (1.02)  2.71 (1.13) | 0.22 (0.40)  1.10 (0.98)  1.66 (1.18)  2.04 (1.19)  2.01 (1.09)  2.16 (1.02)  2.77 (0.95)  3.15 (1.01) | **0.006**  0.052  **0.049**  0.168  0.945  0.684  0.321  **0.011** |
| **Protein-energy ratio (PER)** (g protein/100kcal), mean (SD)  1  3  5  7  10  14  21  28 | 1.23 (1.98)  2.44 (1.86)  2.56 (1.54)  2.57 (1.23)  2.32 (1.03)  2.33 (0.94)  2.50 (0.69)  2.55 (0.67) | 1.91 (2.60)  2.88 (1.73)  3.00 (1.28)  2.79 (0.95)  2.34 (0.91)  2.33 (0.89)  2.47 (0.70)  2.45 (0.80) | 0.90 (1.49)  2.21 (1.89)  2.34 (1.61)  2.46 (1.33)  2.32 (1.09)  2.33 (0.96)  2.51 (0.70)  2.60 (0.60) | **0.002**  **0.027**  **0.008**  0.092  0.909  0.970  0.790  0.183 |

*Median values are additionally presented at 7, 14, 21 and 28 days due to non-normal distributions, as some infants were clinically unwell (e.g. sepsis or abdominal distension) and receiving full or partial intravenous fluids on the day of data collection.

Table ST2. Multiple linear regression of the ten independent variables included in the analysis of energy intake at 28 days of life.

| Source | SS | df | MS |
| --- | --- | --- | --- |
| Model | 18629.6334 | 10 | 1862.9633 |
| Residual | 73271.5926 | 126 | 581.5205 |
| Total | 91901.2260 | 136 | 675.7443 |

Number of obs	137

F (10, 126)	3.20

Prob > F	0.0011

R-squared	0.2027

Adj R-squared	0.1394

Root MSE	24.1150

| **No.** | **Factors associated with energy intake at 28 days of life** | **Coef** | **Std Err** | **t** | **P>\|t\|** | **95% CI** |
| --- | --- | --- | --- | --- | --- | --- |
| 1 | Birthweight | -0.0261 | 0.0152 | -1.72 | 0.088 | -0.0563 to 0.0039 |
| 2 | Gestational age | -1.9799 | 2.0321 | -0.97 | 0.332 | -6.0014 to 2.0416 |
| 3 | Duration of CPAP | -0.3166 | 0.2297 | -1.38 | 0.171 | -0.7713 to 0.1380 |
| 4 | Duration of ventilation | 0.0291 | 0.2712 | 0.11 | 0.915 | -0.5076 to 0.5660 |
| 5 | Admission temperature | 1.7000 | 2.1723 | 0.78 | 0.435 | -2.5989 to 5.9989 |
| 6 | CRIB II score | -2.7464 | 1.6663 | -1.65 | 0.102 | -6.0441 to 0.5512 |
| 7 | **BPD** | -11.7310 | 5.7383 | -2.04 | **0.043** | -23.0870 to -0.3751 |
| 8 | **NEC** | -33.2648 | 12.1075 | -2.75 | **0.007** | -57.2253 to -9.3043 |
| 9 | Sepsis | -5.8792 | 7.4009 | -0.79 | 0.428 | -20.5254 to 8.7669 |
| 10 | sIVH | -15.4666 | 9.9347 | -1.56 | 0.122 | -35.1271 to 4.1938 |
|  | Constant | 168.9895 | 126.864 | 1.33 | 0.185 | -82.0706 to 420.0497 |

Note: sIVH (IVH grade 3 and 4)

Table ST3. Assessment of interaction terms and multicollinearity among factors associated with total energy intake at 28 days of life.

Table ST3A. Checking for interactions.

| **Variable** | **B** | **Std err** | **T** | **P>[t]** | **95% Conf Interval** | |
| --- | --- | --- | --- | --- | --- | --- |
| Birthweight | -0.0266 | 0.0152 | -1.75 | 0.083 | -0.0568 | 0.0036 |
| BPD | -11.5630 | 5.8405 | -1.98 | 0.050 | -23.1222 | -0.0039 |
| NEC | -26.3808 | 13.2246 | -1.99 | 0.048 | -52.5539 | -0.2076 |
| BPD*NEC^a^ | -2.6304 | 28.1358 | -0.09 | 0.926 | -58.3146 | 53.0538 |
| _cons | 184.9702 | 126.9265 | 1.46 | 0.148 | -66.2331 | 436.1734 |

Note: ^a^There was no interaction between BPD and NEC.

Table ST3B. Checking for multicollinearity – variance inflation factors (VIF) for independent variables.

| **Variable** | **VIF** | **1/ VIF** |
| --- | --- | --- |
| CRIB II score | 6.02 | 0.1662 |
| Gestational age | 4.04 | 0.2475 |
| Birthweight | 2.86 | 0.3502 |
| Duration of ventilation | 2.14 | 0.4682 |
| Duration of CPAP | 1.87 | 0.5247 |
| BPD | 1.84 | 0.5435 |
| Admission temperature | 1.61 | 0.6221 |
| Severe IVH grade≥3 | 1.28 | 0.7822 |
| Sepsis | 1.26 | 0.7948 |
| NEC | 1.21 | 0.8234 |
| Mean VIF | 2.41 |  |

Note: All VIFs for the independent variables were <10, indicating no multicollinearity.
